# Structural Mechanism of *N*-Methyl-D-Aspartate Receptor Type 1 Partial Agonism

**DOI:** 10.1371/journal.pone.0047604

**Published:** 2012-10-15

**Authors:** Mikko Ylilauri, Olli T. Pentikäinen

**Affiliations:** Computational Bioscience Laboratory, Department of Biological and Environmental Science & Nanoscience Center, University of Jyväskylä, Jyväskylä, Finland; Emory University, United States of America

## Abstract

*N*-methyl-D-aspartate (NMDA) receptors belong to a family of ionotropic glutamate receptors that contribute to the signal transmission in the central nervous system. NMDA receptors are heterotetramers that usually consist of two GluN1 and GluN2 monomers. The extracellular ligand-binding domain (LBD) of a monomer is comprised of discontinuous segments that form the functional domains D1 and D2. While the binding of a full agonist glycine to LBD of GluN1 is linked to cleft closure and subsequent ion-channel opening, partial agonists are known to activate the receptor only sub-maximally. Although the crystal structures of the LBD of related GluA2 receptor explain the mechanism for the partial agonism, structures of GluN1-LBD cannot distinguish the difference between full and partial agonists. It is, however, probable that the partial agonists of GluN1 alter the structure of the LBD in order to result in a different pharmacological response than seen with full agonists. In this study, we used molecular dynamics simulations to reveal an intermediate closure-stage for GluN1, which is unseen in crystal structures. According to our calculations, this intermediate closure is not a transient stage but an energetically stable conformation. Our results demonstrate that the partial agonist cannot exert firm GluN1-LBD closure, especially if there is even a small force that disrupts the LBD closure. Accordingly, this result suggests the importance of forces from the ion channel for the relationship between pharmacological response and the structure of the LBD of members of this receptor family.

## Introduction

**Figure 1:**
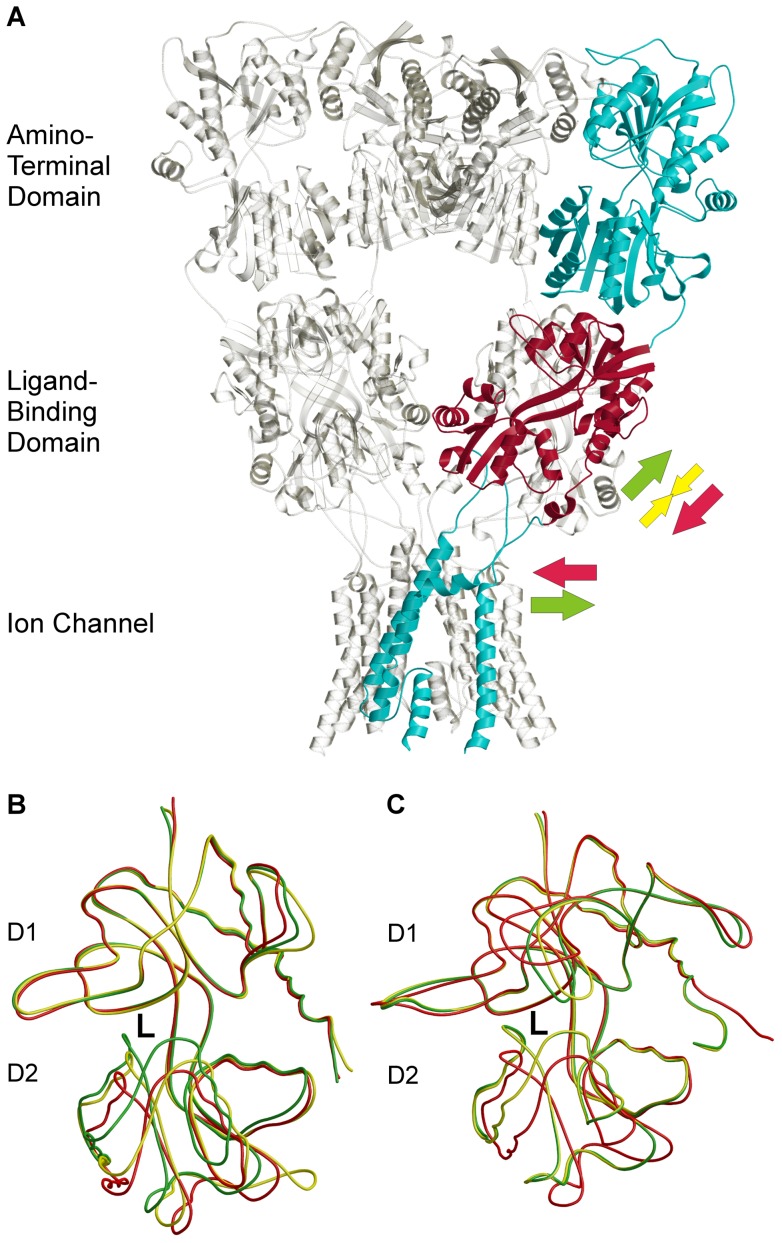
The crystal structure of iGluRs. (A) The crystal structure of GluA2 shows that it functions as a tetramer and (B) that the closure of the LBD determines the pharmacological behavior of GluA2. (C) On the contrary to GluA2, partial agonism of the NMDA receptors is ambiguous. In (A), one LBD (from PDB: 3KG2) is highlighted in red. The arrows depict the potential forces that occur during full agonist binding (green), partial agonist binding (yellow), and closure of the ion channel (red). In (B) and (C), superimposed structures with full agonist (green), partial agonist (yellow), and antagonist (red) are shown. Ligand binding site between domains D1 and D2 is depicted as letter L. Structures (PDB-codes) used are 3KG2 [7] in (A), 1FTJ, 1FTK, and 1FTL [13] in (B) and 1PB7, 1PB9, and 1Y1M [11], [12] in (C).

N-methyl-D-aspartate receptors (NMDARs) belong to a family of ionotropic glutamate receptors (iGluRs) that contribute to signal transmission in the central nervous system [1]. NMDARs play crucial roles in learning and synaptic plasticity, for example [2], [3], [4]. All the iGluRs have been implicated in various diseases, especially neurological disorders. Disease states linked to NMDARs include Parkinson's disease, schizophrenia and stroke, among others [5], [6]. Similar to GluA2 (Fig. 1A), NMDAR probably is a heterotetramer that usually consists of two GluN1 (NMDA-R1) and GluN2 (NMDA-R2) monomers [7]. The functional heterogeneity of NMDARs arises from a wide variety of GluN2 subunits (for a recent review, see [8]). The ligand-binding domain (LBD) of iGluRs is comprised of discontinuous segments that form the functional domains 1 and 2 (D1 and D2) [9]. Although the recombinant LBD forms only part of the iGluR monomer, it shows a similar ligand-binding affinity to that of wild-type receptors [10], [11], [12]. Thus, this domain has been widely applied in crystallography, for example [11], [12], [13], [14], [15], [16] (Fig. 1B–C). Full agonists provoke full LBD closure, leading to opening of the ion channel [13]. In contrast to the AMPA-selective glutamate receptor 2 (GluA2; GluR2) where partial agonists wedge the LBD into a moderately closed state [13], [17] (Fig. 1B), the crystal structures of GluN1 imply that the partial agonists induce full receptor closure [11] (Fig. 1C), pointing to a different mechanism. This view was supported by a recent study [18] that used luminescence resonance energy transfer (LRET) to measure the extent of cleft closure in GluN1. No difference was found between the closure stages of full or partial agonist bound GluN1-LBD. Interestingly, however, in the same study, GluN2-LBD exhibited an intermediate cleft closure when bound to a partial agonist.

In addition to many crystallization studies, the ligand binding and closure of the iGluR-LBD have been explored using various experimental methods, including electrophysiology [12], [19], [20], fluorescence resonance energy transfer [21], and radioligand binding [16]. In addition to these experimental approaches, several recent studies have also exploited sophisticated computational methods to examine the structure and function of iGluRs. In particular, molecular dynamics (MD) simulations have been utilized to study the motion of receptor and ligand-receptor interactions occurring in solvent [22], [23]. For example, the role of water molecules inside the ligand-binding cleft [24], the pharmacology of novel ligands [25], and the subtype selectivity of antagonist ligands [26] have been studied with the help of this *in silico* method. However, closing an open-cleft receptor with a bound ligand has been reached computationally thus far only when exploited with biased MD simulations, for example the umbrella sampling method [27].

The antagonism of NMDA receptors has been widely studied for possible treatment of many neurological disorders [5], [28]. However, it has been proposed that partial agonists could be more advantageous as therapeutics because of their capability to permit some level of normal synaptic transmission while simultaneously suppressing excessive activation [29], [30], [31]. In fact, it has recently become evident that GluN1-specific partial agonists could be used to treat autism, for example (see [32] for review). However, although a growing number of studies concerning partial agonism of NMDA receptors have been published (see for example [12], [20], [33], [34]), only a few have examined the structure and motion of the LBD and its interactions with the ligand at the atomic level [22], [35], [36].

We have previously shown in MD simulations that the GluN1-LBD is able to adjust to more open conformations than crystallization studies have shown [36]. In addition, we have suggested that the stability of the cleft closure is associated with partial agonism. Incomplete closure of the GluN1-LBD with a bound partial agonist is not only interesting but also highly important pharmacologically. Indeed, it has been shown that the intrasubunit movements at linkers between LBD and transmembrane (TM) region are tightly coupled across the four subunits of NMDAR [37]. Thus, the binding of partial agonist molecules to two GluN1 subunits of the tetrameric receptor, which leads to incomplete closure of the LBD, would prevent full ion channel opening despite simultaneous full agonist binding to two GluN2 subunits.

In the present study, various computational methods were utilized in order to obtain a detailed view of the interactions taking place when a partial agonist binds in the GluN1-LBD. We performed steered molecular dynamics (SMD) simulations to study the firmness of full or partial agonist bound GluN1 structures. We also used constraint-free MD simulations to study the different closure stages and critical interactions of GluN1 with bound ligand. In addition, ligand-binding energetics with different closure stages of GluN1 were measured using the molecular mechanics generalized Born/surface area (MMGB/SA) method [38], [39].

## Results and Discussion

We have previously shown that full agonists keep the iGluR-LBD closed, whereas partial agonists destabilize the cleft closure [36]. To examine LBD closure in detail, we measured the distances between various atoms from MD and SMD trajectories to investigate the interactions that take place between the ligand and GluN1 during the closure of the GluN1 ligand-binding cleft. In addition, visual inspection of the LBD in snapshot structures of MD aided the evaluation of changes in the conformations of amino acids participating in the ligand binding.

**Figure 2:**
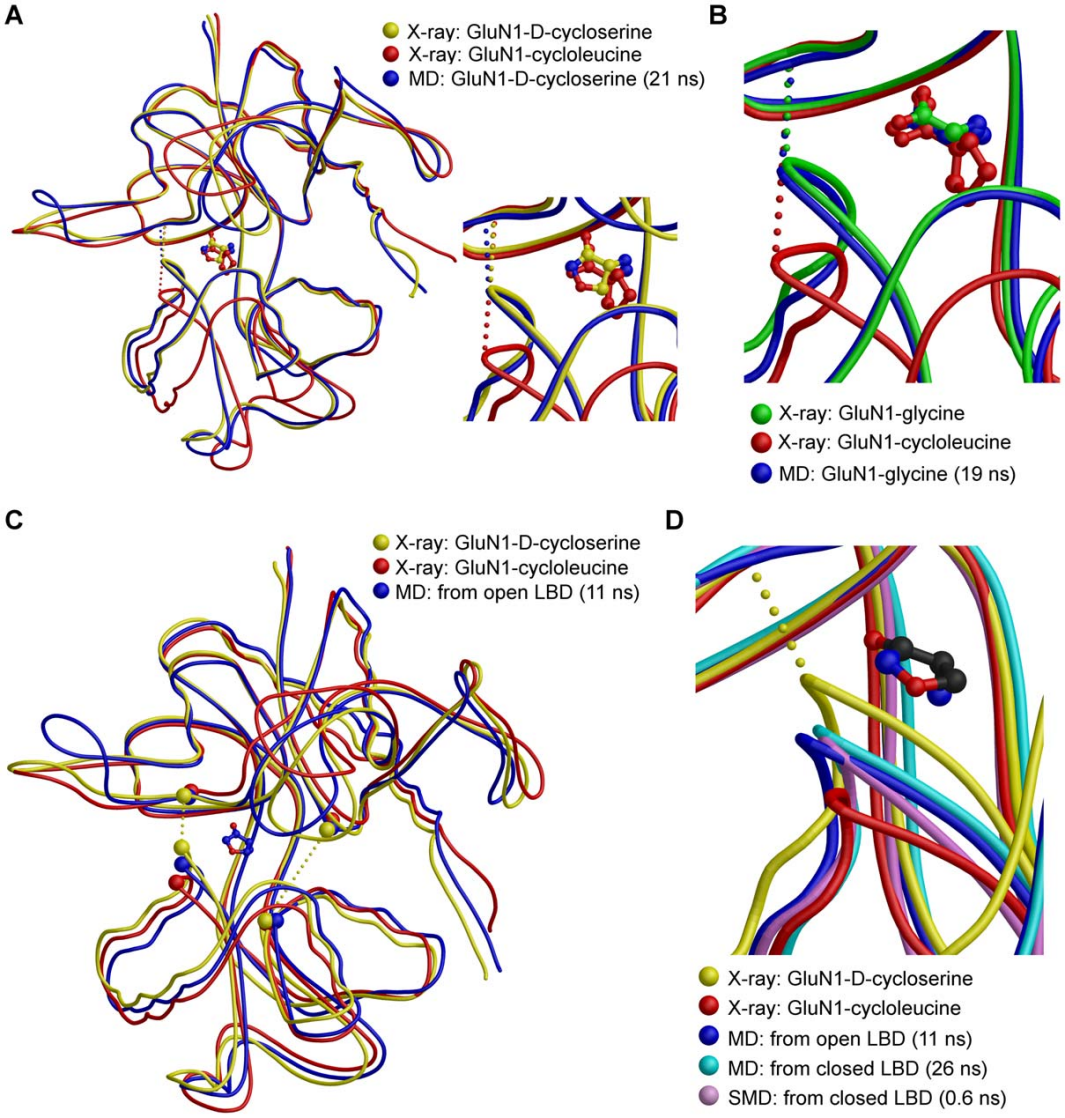
MD and SMD simulations of ligand-bound GluN1-LBD. Free MD simulations indicate that (A) D-cycloserine and (B) glycine bound to open-cleft GluN1 (from PDB: 1Y1M) can close the LBD between D1 and D2, as seen in the crystal structures. (C) Contrary to crystal structures, a stable intermediate closure stage is seen in GluN1-LBD with bound partial agonists. Superimposition of a snapshot from a D-cycloserine simulation in Fig. 3A (blue line) with crystal structures of the same ligand (PDB: 1PB9) and antagonist ligand cycloleucine (from PDB: 1Y1M) is shown. Cα atoms of IHB residues (Gly485 and Gln686), as well as of residues Gln405 and Ala715, are depicted as CPK, and dotted lines represent the distances measured to study the closure of the cleft. (D) A close-up of the intermediately closed GluN1-D-cycloserine structures in free MD simulations – starting from both closed and open-cleft structures – as well as in SMD simulation starting from a closed-cleft structure (6 pN, blue line in Figure S2). Crystal structures of GluN1 with bound D-cycloserine (from PDB: 1PB9) and cycloleucine (from PDB: 1Y1M) are superimposed for comparison. Dotted lines in (A), (B), and (D) represent the IHB distance between Gly485^N^ and Gln686^O^, which is an efficient indicator of cleft closure.

In constraint-free MD simulations, a full agonist, glycine, and partial agonists D-cycloserine, 1-aminocyclopropane-1-carboxylic acid (ACPC), and 1-aminocyclobutane-1-carboxylic acid (ACBC) were inserted into the open-cleft conformation of GluN1-LBD. In MD simulations, the smaller ligands glycine, D-cycloserine, and ACPC induced closure of the cleft (Figs. 2A–B and S1A), whereas ACBC, which has a bulkier structure, did not (Fig. S1B). Using glycine, this closure was sometimes obtained after 15 ns (Fig. 2B). However, in some simulations, closure occurred only after 120 ns. For D-cycloserine and ACPC, the closure times for GluN1-LBD were 19 ns and 6 ns, respectively (Figs. 2A and S1A). However, this result was not obtained regularly with either partial agonist in up to 127 ns simulations using the same setup. In this study, for the first time, the ligand-induced iGluR-LBD closure was repeatedly obtained in a constraint-free MD simulation with no artificial modifications (e.g., umbrella sampling, temperature shift, etc.). It is most likely that the closure of the GluN1 cleft is easier to obtain in a constraint-free MD simulation than closure of the other iGluRs because the solvent molecules are not as crucial in the ligand-binding process. The easier closure of GluN1 with bound agonist ligand is thus likely due to the lack of polar interactions between bound ligand and the D2, which is the case with other iGluR subtypes.

**Figure 3:**
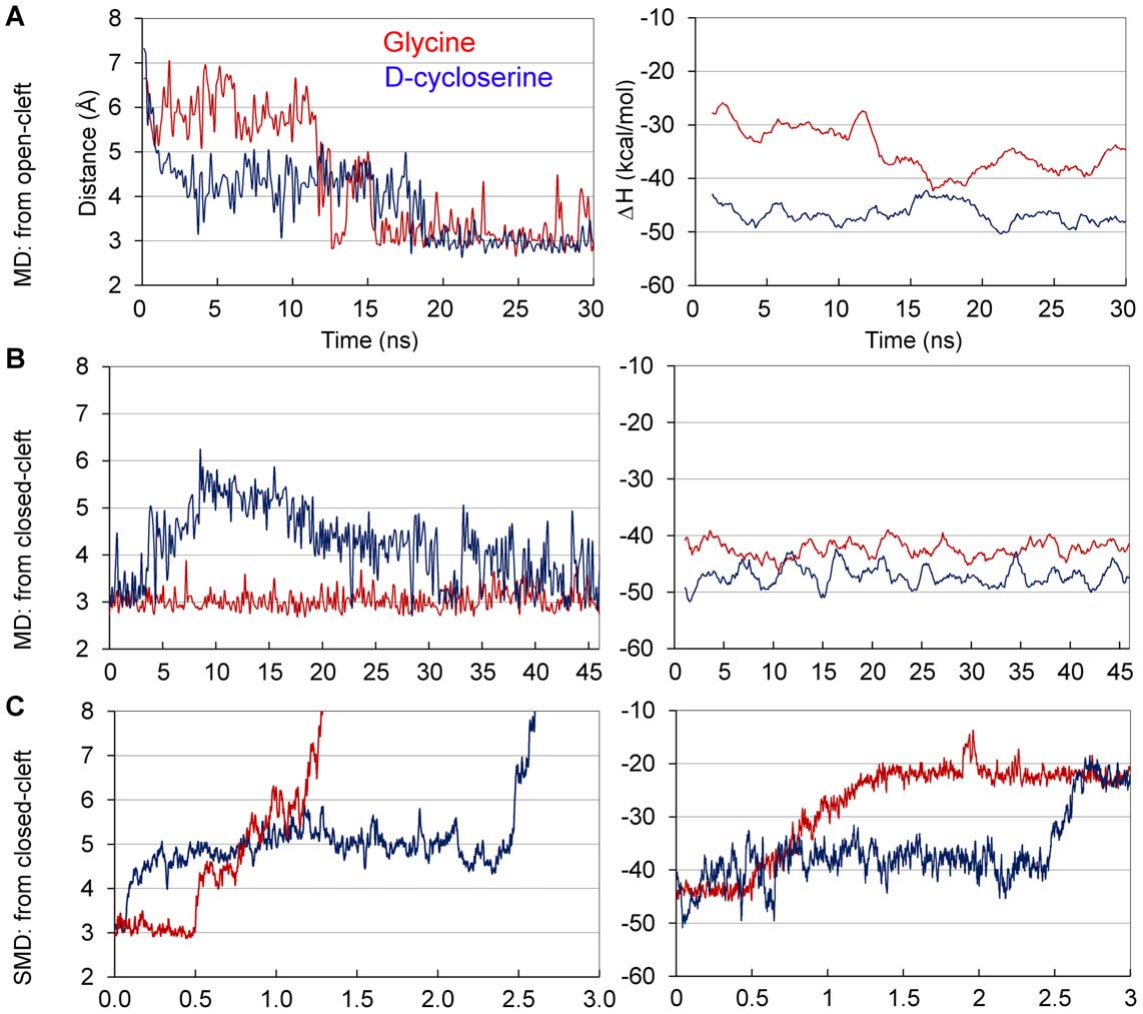
Relationship of GluN1-LBD closure and ΔH in ligand binding. Free MD simulations starting from (A) open and (B) closed LBD, and (C) SMD simulations (9 pN) from a closed LBD. The distances (left panel) are IHB distances (Gly485^N^-Gln686^O^). Corresponding binding enthalpies (ΔH) from the simulations are shown in the right panel. Results from all the SMD simulations performed are shown in Figure S2.

It is interesting to note that contrary to simulations with a full agonist, with all partial agonists a relatively stable intermediately closed conformation stage of the LBD appears to exist (Fig. 2C). In each partial agonist studied, this phase extended over a period of several nanoseconds, up to 16 ns in one of the MD simulations with D-cycloserine (Figs. 3A and S1). In the closed conformation, an interdomain hydrogen bond (IHB) exists between Gly485^N^-Gln686^O^. The IHB has previously been shown to be an efficient indicator of cleft closure [36]. However, in the intermediate closure, this distance is clearly longer (4-5 Å with D-cycloserine and 5–6 Å with ACPC), albeit not as much as in the crystal structure of the GluN1-cycloleucine complex (7.1 Å). Interestingly, in this study, the intermediate closure obtained from an open-cleft conformation is very similar to that obtained from a closed-cleft conformation in the GluN1–D-cycloserine simulation (Fig. 3B) [36]. In addition to the intermediate closure with IHB distance of 4–5 Å, with ACBC, another intermediate stage was seen in some simulations at approximately 5.5 Å (Fig. S1B). The intermediate closure was not observed with full agonist glycine, regardless of the starting conformation (Fig. 3A–B). To investigate the effect of the observed intermediate closure on ligand positioning, we measured root-mean-square deviation (RMSD) values in the MD trajectories. According to average values calculated over intermediate and fully closed stages, RMSD for partial agonists remained stable. For example, in the D-cycloserine simulation of the open-cleft structure (Fig. 3A), an average value of RMSD (fit to previous frame) was 0.98±0.26 for both 3–18 and 20–30 ns time ranges. These results indicate that the closing of the open-cleft LBD does not affect the fluctuation of the ligand conformation. However, in the open-cleft stage (0–3 ns), the average RMSD value was slightly higher (1.14±0.27), indicating that the ligand is more unrestrained to move in the cleft.

In addition to distances, we also studied the IHB angles of N-H-O and C-O-H in the MD simulation trajectories. IHB angles form between the main chain atom H (bonded to N) of Gly485 and O (bonded to C) of Gln686. Optimal angles for the triangles N-H-O and C-O-H are approximately 180° and 120°, respectively. The measured angles in both full agonist and partial agonist bound GluN1-LBD simulations deviated from these optimal values, yet they remained constant in the normal range. For D-cycloserine, the average angles of N-H-O and C-O-H when binding cavity was closed were 150±12 degrees and 155±12 degrees, respectively. For glycine, the same average angles were 148±12 for N-H-O and 161±10 for C-O-H. It must be noted that the corresponding angles in the crystal structures also differ somewhat from the optimal angle values: For glycine, the angles of N-H-O and C-O-H are 165.9 and 157.9, respectively. For D-cycloserine, the equivalent angles in an X-ray structure are 163.9 and 158.3.

**Figure 4:**
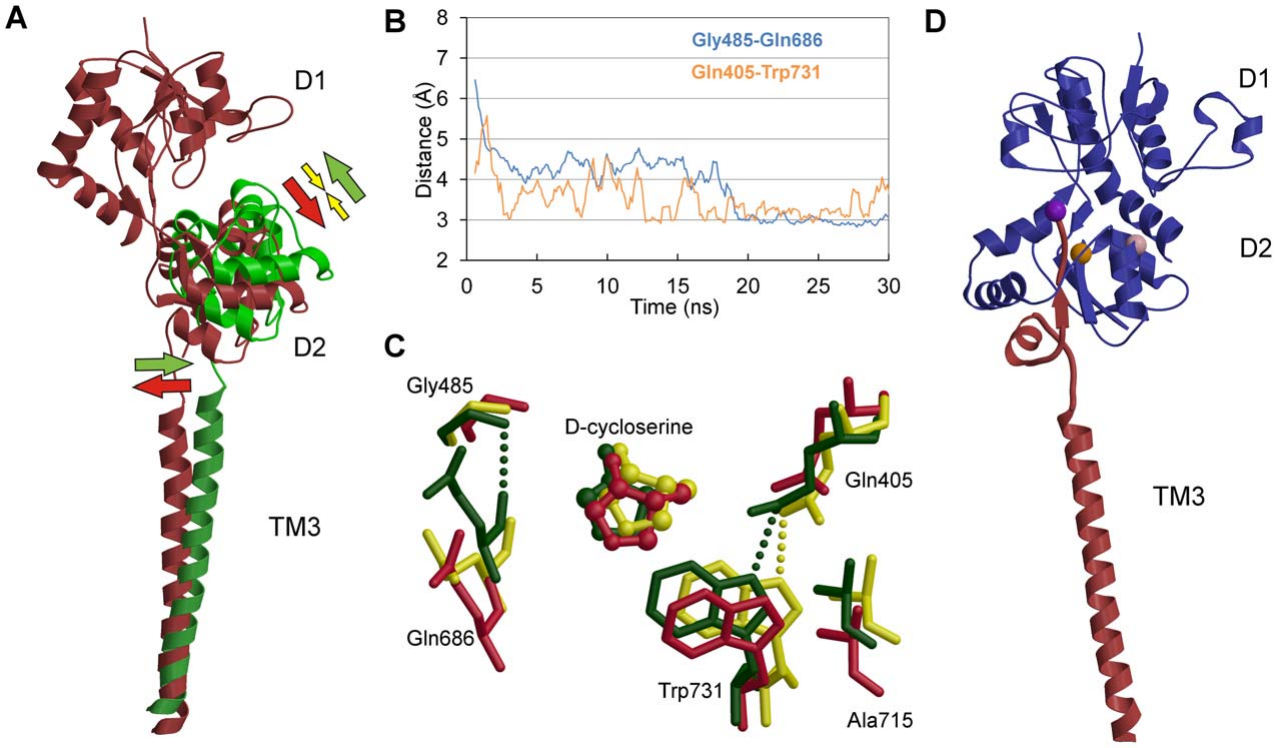
Closure mechanism of GluN1-LBD and connection to transmembrane domain. (A) Model showing the hypothesized conformational changes taking place at LBD and TM domain in binding of either an agonist or antagonist to cleft between D1 and D2. Agonist and antagonist bound models are colored green and red, respectively. Colored arrows depict the hypothesized forces affecting the conformation of the domains (full agonist in green, partial agonist in yellow and antagonist in red). (B) Distance measurements of Gly458^N^-Gln686^O^ and Gln405^OE1^-Trp731^NE1^ from D-cycloserine bound open-cleft GluN1-LBD taken from a constraint-free MD simulation trajectory. Comparison of the two distances reveals that there is a difference in the swiftness of closure of the LBD at various sides of the binding cleft. In addition, the intermediate closure is not seen ubiquitously at the binding cavity. In (C), superimposed structures are taken from the trajectory of the simulation in (B). The starting structure, cycloleucine-bound open-cleft GluN1 (PDB: 1Y1M), is colored red. Snapshots from intermediately closed (yellow) and fully closed (green) LBD are taken at time steps of 13 and 19 ns, respectively. In (D), part of an iGluR monomer (from GluA2 structure, PDB: 3KG2) show that the IHB is directly linked to M3. The purple ball represents the location of Gly458, which is the IHB-residue at D2 side of GluN1-LBD. Locations of Trp731 and Ala715 are depicted as orange and pink balls, respectively.

To mimic the forces that likely apply to GluN1-LBD upon closure of the ion-channel (Figs. 1A and 4A: red arrows), we used SMD simulations with a constant force (6–10 pN) that was applied to C^α^ atoms of D2 while D1 was fixed. The direction of the force, which was defined by the vector that links the center of mass of C^α^ atoms of D1 and D2, simulated well the proposed force that was directed on the LBD and which induced the opening of the ligand-binding cleft (Fig. 4A, red arrows show the hypothetical movement of the ion channel forming transmembrane helix 3 (M3) that would lead into opening of the ion channel). These simulations revealed that the ligand-binding cleft closes more firmly with full agonists than partial agonists. In most cases, a glycine-bound structure remained closed even in a simulation with 8 pN force, although in some simulation runs the structure stayed shut at as high as 10 pN force. In contrast, the IHB in partial agonist simulations was broken readily with weaker forces, even at 6 pN (Fig. S2).

It is remarkable that in SMD simulations, with all the partial agonists the structures settled on the same intermediate closure as seen in free MD simulations (Figs. 3C and S2). In some of the 3 ns runs, when this closure stage was reached, it remained stable throughout the rest of the simulation. In some other runs, especially with higher forces, the LBD was first settled on the intermediate closure stage but later was fully opened. Similar to constraint-free MD simulations, this stage was not seen in any of the SMD runs with full agonist glycine-bound GluN1-LBD (Figs. 3C and S2). The average IHB distances from SMD simulations were calculated for all the partial agonists studied at the intermediate closure stage. With bound D-cycloserine, GluN1-LBD settles to an average of 4.8±0.5 Å distance when intermediately closed. Similar closure degrees for ACPC and ACBC are 5.4±0.2 and 5.5±0.2 Å, respectively. It is difficult to extract definitive values due to the nature of the method and for the fact that the exact determination of the start and end points of the intermediate closure-stage is awkward. However, a rough comparison of the agonist efficacies of different ligands to experimental data ([12] [40]) suggests that the average closure degrees from MD simulations correlate with the experimental results: Priestley et al. (1995) [40] showed that D-cycloserine activates GluN1 by 88%±4 and ACBC by 33±7% compared to full agonist glycine, while Inanobe et al. (2005) [12] demonstrate ACPC and ACBC to have 80% and 42% activation, respectively. Thus, our results of distance calculations return, in some extent, these previous experimental findings; the smaller the IHB distance in intermediate closure, the more effective the ligand (Table 1).

**Table 1:** Average IHB distance and ΔH from SMD simulations compared to experimentally obtained efficacies and EC_50_ values for various GluN1 agonists.

	Distance/Efficacy		Energy/Potency	
Ligand	IHB (Å)^a^	Efficacy (%)^b^	ΔH (kcal/mol)^c^	EC_50_ (µM)^d^
Glycine	2.9	100	−43.9	0.72
D-cycloserine	4.8	88	−38.4	8.2
ACPC	5.4	80	−43.9	0.65
ACBC	5.5	33	−38.7	6.6

a Calculated as average distances between Gly485^N^ and Gln686^O^ at the intermediate closure stage. For glycine, distance is measured from PDB-structure 1PB7.

b Experimental efficacies (from GluN1/GluN2B assemblies) compared to glycine. Data for D-cycloserine and ACBC from [40], ACPC from [12].

c Average ΔH calculated by MMGB/SA from the time-span of intermediate closure. For glycine, ΔH was averaged from the time period of fully closed state.

d EC_50_ data (from GluN1/GluN2B assemblies) obtained from literature: glycine and ACPC from [19], D-cycloserine and ACBC from [35] and [40], respectively.

The mechanism of closure of the LBD was analyzed in MD simulations starting from the open-cleft LBD. The distances of several atoms from MD trajectories were measured at different sides of the binding cavity. In addition, snapshots extracted from the trajectories were visually inspected. The distance measurements showed that closure does not occur similarly and simultaneously in every part of the cavity. This was most evident when the distance between Gln405 and Trp731 from D1 and D2, respectively, was compared to IHB-distance (Gly485-Gln686) in MD simulations with partial agonists (Figs. 4B and S3). These two pairs are situated at separate sides of the cleft, IHB residing near helix F and loop 2 and Gln405-Trp731 between helices H and I. Although they both form a hydrogen bond as the binding cavity closes, Gln405-Trp731 bonding occurs much more rapidly. The swift closure at this part of the cavity is followed by a slower closure at the other end, which was seen in the IHB distance curve. A similar difference in the closure mechanism was previously seen in simulations of GluK1-LBD using partial agonist 9-deoxy-neoDH [23]. Interestingly, it appears that the intermediate closure stage has not been seen in the cleft area near helices H and I. In addition to the distance measurements, this is also evident when the superimposed snapshot structures are examined (Fig. 4C). Although an intermediate stage is clearly seen in residues forming the IHB, the area at the other side of the cavity has only two distinct closure stages. This might explain the previous results obtained with LRET, in which no intermediate closure of GluN1-LBD was seen with partial agonist when the distance was measured from Ala715 to Thr396 [18]. Because an isolated LBD was used in our MD studies, the N-terminal Thr396 is reasonably free to move during the simulations. Accordingly, it was not practical to measure this same distance in our study. However, Ala715, residing in helix H and depicted in Figure 4C, clearly shows movement similar to Trp731, which has only two distinct closure stages. The difference in the closure mechanism of LBD at separate sides of the cleft might be explained by taking into account how the LBD is linked to TM domain. M3, and especially the M3-S2 linker between TM and domain 2 of LBD, are presumed crucial in the gating process [7]. M3 helices form the ion pore in tetrameric iGluR [7], and M3-S2 likely transmits the conformational dynamics between TM and LBD. As shown in Figure 4D, the region of D2 near the IHB residues is closely linked to M3. Accordingly, any force directed on LBD from TM readily affects the conformation of this region of the LBD. On the contrary, Trp731 and Ala715 are not directly linked to TM (Fig. 4D). This possibly explains why the intermediate closure is seen only at some parts of the binding cleft. Additional explanation for the difference in the D1–D2 interaction at different parts of the cavity might be that while the IHB forms between main-chain atoms, the bond between Gln405 and Trp731 utilizes atoms of amino acid side-chains. Thus, the bond involving side chain atoms has more freedom to adapt to small movements at the D1–D2 interface compared to more restricted bond between main-chain N and O atoms. This difference between various parts of the cleft is analogous to that seen in the structure of GluA2 with bound kainate [9]. Earlier, it has been suggested that the movements at the hinge-region and the small movements of the Trp731 side-chain play a role in the mechanism of partial agonism [12]. However, our results indicate that there is no ligand-dependent motion at the hinge-region, and while the Trp731 indole ring may be able to slightly change its conformation depending on the ligand, the above mentioned hydrogen bond to Gln405 remains formed with both full and partial agonists. Thus, no intermediate closure is seen at that part of the ligand-binding cavity.

To study the energetic basis of the closure, ΔH was estimated from the MD and SMD trajectories by the MMGB/SA method. In the MD simulations of the open-cleft LBD with bound glycine, there was a clear decrease (10 kcal/mol) in energy when the cleft closed (Fig. 3A, 16 ns). In the simulations of the closed receptor, ΔH was similar throughout the simulation (Fig. 3B), indicating that the interactions in the GluN1-LBD complex did not change. In the SMD simulations, when the cleft opened, ΔH of glycine binding increased (Fig. 3C). Thus, MMGB/SA calculations indicated that the full agonist favors the closed LBD. In the MD simulation of open-stage GluN1-LBD with bound D-cycloserine, the ΔH was similar in both intermediate (Fig. 3A, 3–18 ns) and closed stages (Fig. 3A, 18–30 ns). This was more apparent in the MD simulation starting from the closed-stage LBD (Fig. 3B): the D-cycloserine-complex opened and remained at the intermediate closure before closing again at a later stage. However, the level of the ΔH did not shift substantially during these changes. In the SMD simulation, the ΔH increased slightly (3–5 kcal/mol) when the LBD opened to the intermediate closure (Fig. 3C). This increase could be explained by the fact that exerting a constant force to pull the D2 affects the binding conformation of D-cycloserine. With ACPC and ACBC, a similar trend was seen in SMD simulations: when the LBD opened to an intermediate stage, the ΔH typically increased only negligibly (Fig. S4). When the calculated ΔH values from SMD simulations (glycine: −43.9 kcal/mol; ACPC: −43.9 kcal/mol; D-cycloserine: −38.4 kcal/mol; ACBC: −38.7 kcal/mol), averaged for the time span of intermediate closure state (fully closed state for glycine), are compared to EC_50_ values reported for agonists (glycine: 0.72 µM [19]; ACPC: 0.85 µM [19]; D-cycloserine: 8.2 µM [35]; ACBC: 6.6 µM [40]), a good correlation can be seen (Table 1). To conclude, full closure of the GluN1-full agonist complex is clearly energetically preferred. On the contrary, with partial agonists the complete closure of GluN1-ligand complex is not necessarily energetically preferred, or at least, the difference between fully and partially closed stages is very small. According to our results from SMD with all three partial agonists, any stress on the LBD, such as from the ion-channel, can force the receptor cleft into the intermediate closure stage.

The co-crystal structures of GluN1-LBD with ligands, contrary to other iGluRs, imply that the degree of domain closure is similar with both full and partial agonists [11], [12]. In this study, we showed an intermediate closure stage exists for GluN1 with a bound partial agonist, similar to that reported for the GluA2-kainate complex [13]. This resemblance is apparent when the structures are superimposed (Figs. 2C–D and 4C). In addition to IHB-distance measurements (Fig. 3), the MMGB/SA calculations showed that this intermediate closure is not a transient stage but a stable and energetically favored conformation. As the agonist binds to the LBD, the ion channel opens. However, it also closes rapidly either by opening the LBD after releasing the bound agonist or, in the case of non-NMDA iGluRs, by entering the desensitization state. In other words, the ion channel persists in staying closed, and accordingly, based on our results it could be hypothesized that there is a force directed on the LBD that segregates D1 from D2. This force would transmit from M3 to LBD via the short linker and affects the conformation at the regions of D2 most closely linked to it. Such force from the TM would not be observed when only isolated LBDs are used, which would explain the missing intermediate closure from the crystal structures of partial agonist bound GluN1-LBD. Based on our results, partial agonists probably keep the receptor slightly open, as previously reported for other iGluR subtypes.

## Methods

### Starting structures

The complete structures of GluN1-LBD monomers with D-cycloserine (PDB: 1PB9) [11], ACPC (PDB: 1Y20) [12], ACBC (PDB: 1Y1Z) [12], glycine (PDB: 1PB7) [11], and cycloleucine (PDB: 1Y1M) [12] were built based on the alignment of the correspondent crystal structure and the rat sequence (GRIN1) [41] using MALIGN in BODIL [42] and NEST [43]. Note that monomer structure of GluN1 was used instead of GluN1/GluN2 dimer. This was done due to there are only D1–D1 interactions seen in the crystal structure of the GluN1-GluN2 LBD-dimer (PDB: 2A5T) [15], and because there is as of yet no solved crystal structures of full tetrameric NMDA receptor; thus, it is currently not possible to confirm the actual interactions existing between GluN1 and GluN2.

For parameterization, the 3D structures of ligands were optimized quantum mechanically with GAUSSIAN03 (Gaussian, Inc., Wallingford, CT) at the HF/6-31+G^*^ level using a polarizable continuum model. The RESP method [44] was used to calculate the atom-centered point charges from the electrostatic potentials. TLEAP in Antechamber-1.27 [45] was used to: (1) generate force field parameters; (2) add hydrogen atoms; (3) neutralize the system by adding two chloride ions; and (4) solvate the system with a rectangular box of transferable intermolecular potential three-point (TIP3P) water molecules extending 13 Å in every dimensions around the solute. The dimensions of the water-filled box in simulations starting from open and closed LBD were 86×87×97 Å and 94×86×91 Å, respectively. Number of water molecules in the box was approximately 20,900 in a box with the open-cleft GluN1-LBD and 17,800 with the closed-cleft.

*Constraint-free MD simulations* were performed for the open-cleft structure of GluN1-LBD, taken from the cycloleucine-bound complex (PDB: 1Y1M) [12]. The ligand position was decided based on the superimposition of C^α^ atoms of glycine or D-cycloserine LBD structures with the cycloleucine structure using VERTAA in BODIL [42]. The antagonist ligand was removed and replaced by either glycine or D-cycloserine from their corresponding X-ray structures. The energy minimization and MD simulations of 30–127 ns were performed with NAMD2.6 [46] using AMBER03 force field. The equilibration of the system was performed in three steps: (1) energy minimization of the water molecules, counter-ions and amino acid side-chains (15,000 steps), while the rest of the system was kept constrained at the same time by restraining C^α^ atoms with a harmonic force of 5 kcal mol^−1^ Å^−2^; (2) energy minimization of the whole system without constrains (15,000 steps); and (3) MD simulation run with restrained C^α^ atoms in constant pressure (30,000 steps). Finally, unstrained production MD simulations were performed (30–127 ns). All production simulations were repeated three times. The temperature was kept at 300 K with Langevin dynamics for all non-hydrogen atoms, using a Langevin damping coefficient of 5 ps^−1^. The pressure was kept at 1 atm with Nosé-Hoover Langevin piston [47] with an oscillation time scale of 200 fs and a damping time scale of 100 fs. An integration time step of 2 fs was used under a multiple time stepping scheme [48]. The bonded and short-range interactions were calculated every third step. A cutoff value of 12 Å was used for the short-range electrostatic interactions and van der Waals forces to smoothen the cutoff. The simulations were conducted under periodic boundary conditions, and the long-range electrostatics were counted with the particle mesh Ewald method [49]. The hydrogen bonds were restrained by the SHAKE algorithm [50].

*In steered molecular dynamics (SMD) simulations*, the C^α^ atoms of D1 of GluN1-LBD (Met394-Tyr535 and Gly757-Ser800) were kept fixed while an external force was applied to the center of mass of the C^α^ atoms of D2 (Gln536-Ser756). The direction of the constant force (6–10 pN) was defined by the vector that links the center of mass of C^α^ atoms of D1 and D2. The simulations were performed as with constraint-free simulations, except that the SMD production runs of 3 ns were performed only after 720 ps unrestrained MD simulation, and the time step used in SMD production simulations was 1 fs.

*Trajectory analyses* of MD and SMD simulations were done by extracting snapshots at 360 ps intervals with PTRAJ in ANTECHAMBER 1.27 [45]. Various atom distances and closure angles, at 120 ps intervals, were measured with PTRAJ from amino acid residues in the ligand-binding pocket. RMSD values, fit to previous frame, were extracted from trajectories to study the ligand-positioning. Visual inspection of snapshots was performed with BODIL. A cutoff value of 3.4 Å was used as the upper limit for a hydrogen bonding distance.

*The binding enthalpies (*Δ*H) of ligands* with implicit solvent model were calculated from the MD and SMD trajectories using molecular mechanics generalized Born/surface area (MMGB/SA) method [38], [39] implemented in Amber10 [51]. Changes in the enthalpy were calculated from snapshots taken from the MD complex trajectory at 120 ps intervals.

*Figures* were generated with BODIL v. 0.81 and MOLSCRIPT v. 2.1.2 [52], and rendered with RASTER3D v. 2.7C [53].

*Modeling the hypothetical M3 helix movements upon agonist ligand binding* (Fig. 4A) was made using the following strategy: (1) D1 domain of GluA2-L-glutamate complex (PDB: 1FTJ) was superimposed with the D1 of the full length GluA2 structure (PDB: 3KG2) (2) D2 of another copy of the full length GluA2 structure was superimposed with D2 of GluA2-L-glutamate complex used in the step (1); finally (3), the intracellular end of the M3 helix of the full length GluA2 structure from step (2) was superimposed (while extracellular end was left in the modeled position) with that of the full length GluA2 structure used in the step (1).

## Supporting Information

Figure S1 Constraint-free MD simulations of ACPC and ACBC. Free MD simulations starting from open GluN1-LBD are shown for (A) ACPC and (B) ACBC. IHB distance (Gly458^N^-Gln686^O^) measurement for two representative repeats is shown for both partial agonists. Simulations with bound ACPC show closure of the LBD (dark blue) and the stable intermediate stage (light blue). In simulations with ACBC, two distinct intermediate stages can be seen: one at 4–5 Å (light blue) and another at 5–6 Å (dark blue, starting from approximately 20 ns).
(TIF)

Figure S2 GluN1-LBD opening in SMD simulations. Openings of glycine, D-cycloserine, ACPC and ACBC-bound closed GluN1-LBD in SMD simulations are shown for various external forces. In the simulations, a constant force (6–10 pN) was applied to C^α^ atoms of D2 (Gln536-Ser756) while the C^α^ atoms of D1 (Met394-Tyr535 and Gly757-Ser800) were kept fixed. Three repeats (colored blue, purple and red) are shown for each ligand and force used.
(TIF)

Figure S3 Closure mechanism of glycine and ACPC-bound GluN1-LBD. Distance measurements of Gly458^N^-Gln686^O^ (blue) and Gln405^OE1^-Trp731^NE1^ (orange) from (A) glycine and (B) ACPC-bound open-cleft GluN1-LBD are taken from constraint-free MD simulation trajectories. Similar distance measurements for D-cycloserine bound LBD are shown in Figure 4B.
(TIF)

Figure S4 Calculated binding enthalpies (ΔH) from SMD simulations of ACPC and ACBC-bound GluN1-LBD. Compared to D-cycloserine, a similar trend was seen in SMD simulations of (A) ACPC and (B) ACBC: when the LBD opened to an intermediate stage, the ΔH increased only negligibly. With ACPC, this is seen from 1.5 to 2.5 ns and with ACBC, from 1.0 to 1.8 ns. IHB distance is shown in blue and the ΔH, estimated by the MMGB/SA method, in black.
(TIF)
