# Supplementary figures and images for: Structural Mechanism of N-Methyl-D-Aspartate Receptor Type 1 Partial Agonism
Source: PLoS One. 2012 Oct 15;7(10):e47604. doi: 10.1371/journal.pone.0047604 (PMC3471861; doi:10.1371/journal.pone.0047604)

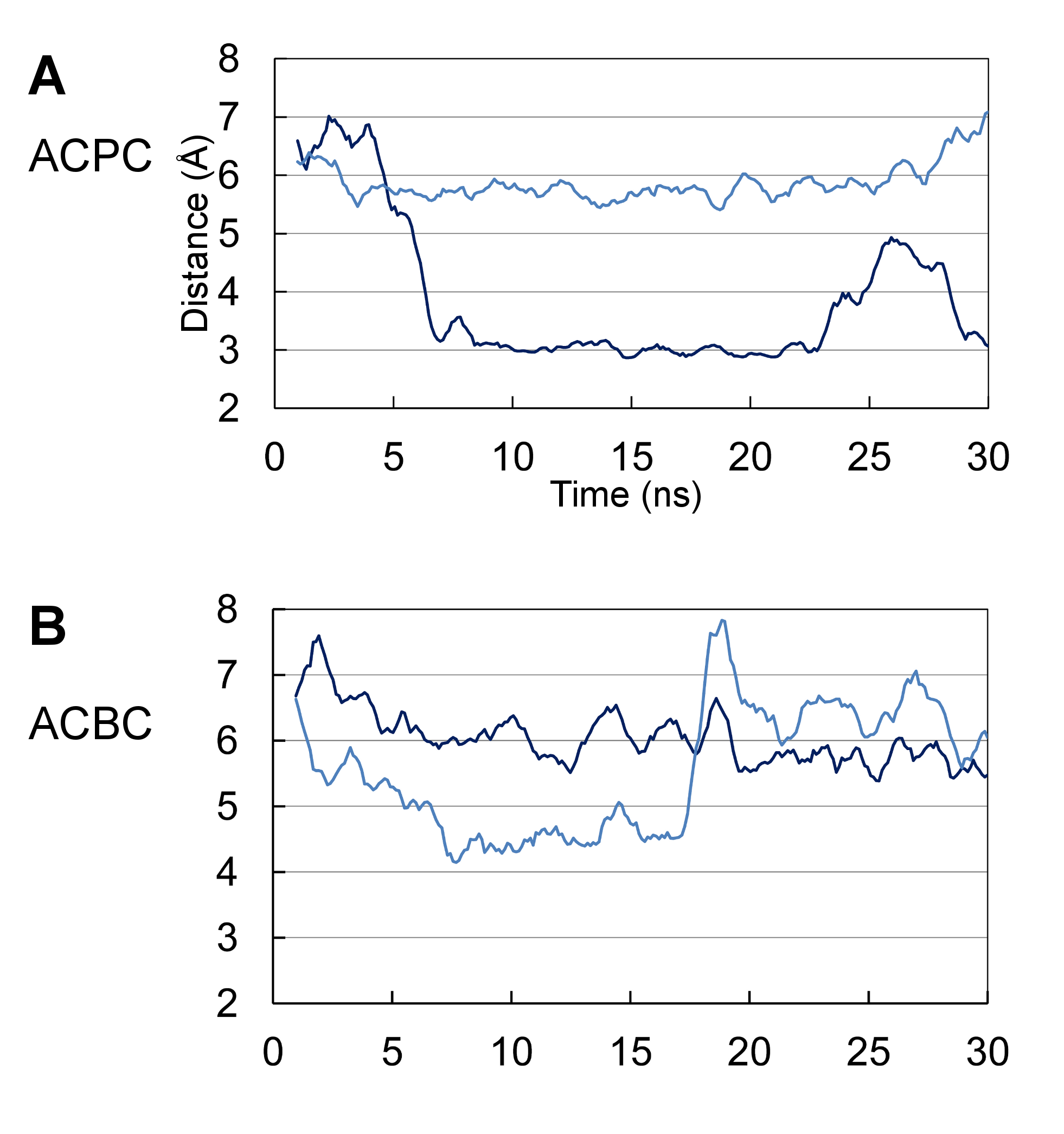

Supplement: Figure S1 — Constraint-free MD simulations of ACPC and ACBC. Free MD simulations starting from open GluN1-LBD are shown for (A) ACPC and (B) ACBC. IHB distance (Gly458N-Gln686O) measurement for two representative repeats is shown for both partial agonists. Simulations with bound ACPC show closure of the LBD (dark blue) and the stable intermediate stage (light blue). In simulations with ACBC, two distinct intermediate stages can be seen: one at 4–5 Å (light blue) and another at 5–6 Å (dark blue, starting from approximately 20 ns). (TIF) [file pone.0047604.s001.tif]

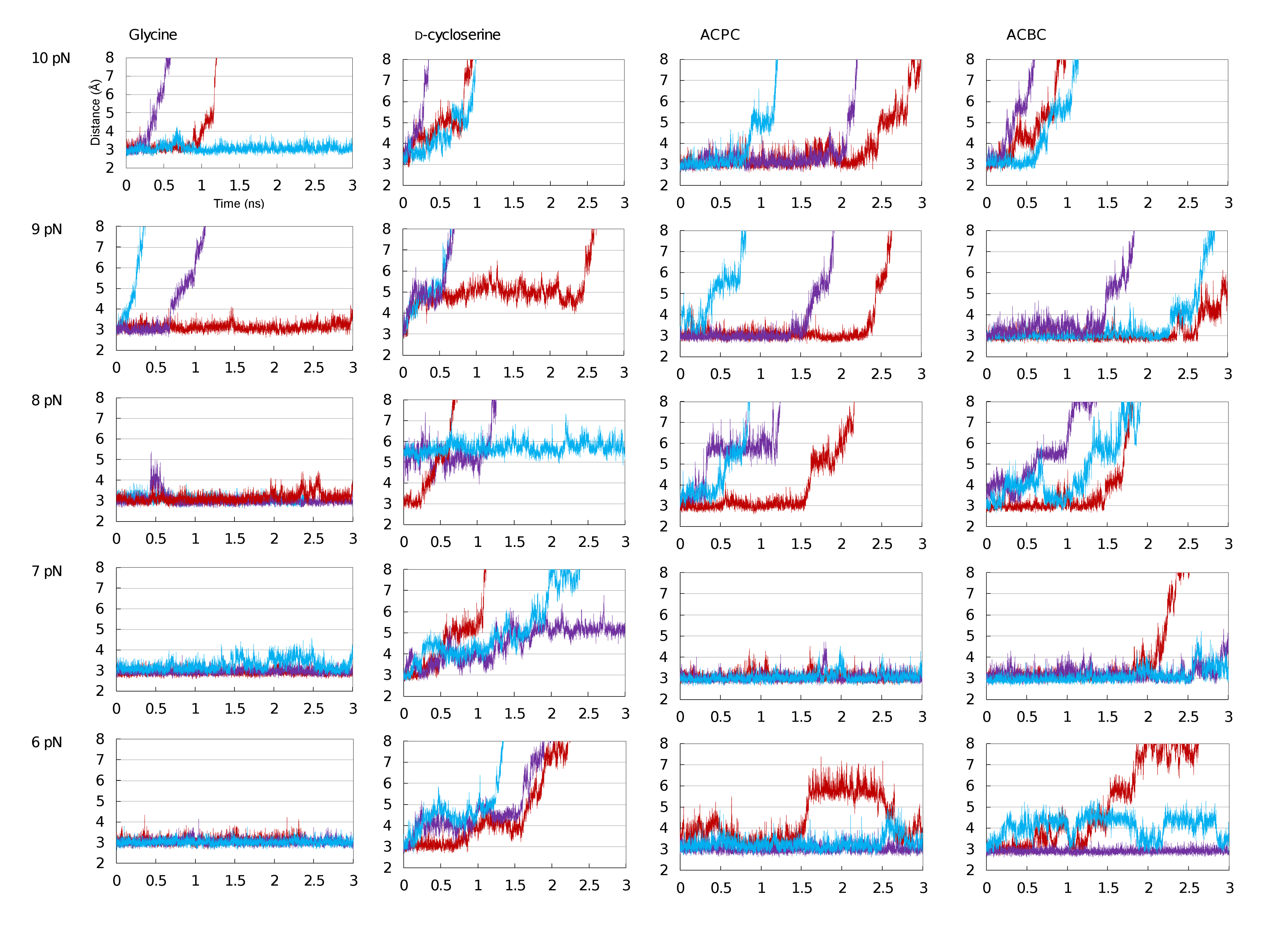

Supplement: Figure S2 — GluN1-LBD opening in SMD simulations. Openings of glycine, D-cycloserine, ACPC and ACBC-bound closed GluN1-LBD in SMD simulations are shown for various external forces. In the simulations, a constant force (6–10 pN) was applied to Cα atoms of D2 (Gln536-Ser756) while the Cα atoms of D1 (Met394-Tyr535 and Gly757-Ser800) were kept fixed. Three repeats (colored blue, purple and red) are shown for each ligand and force used. (TIF) [file pone.0047604.s002.tif]

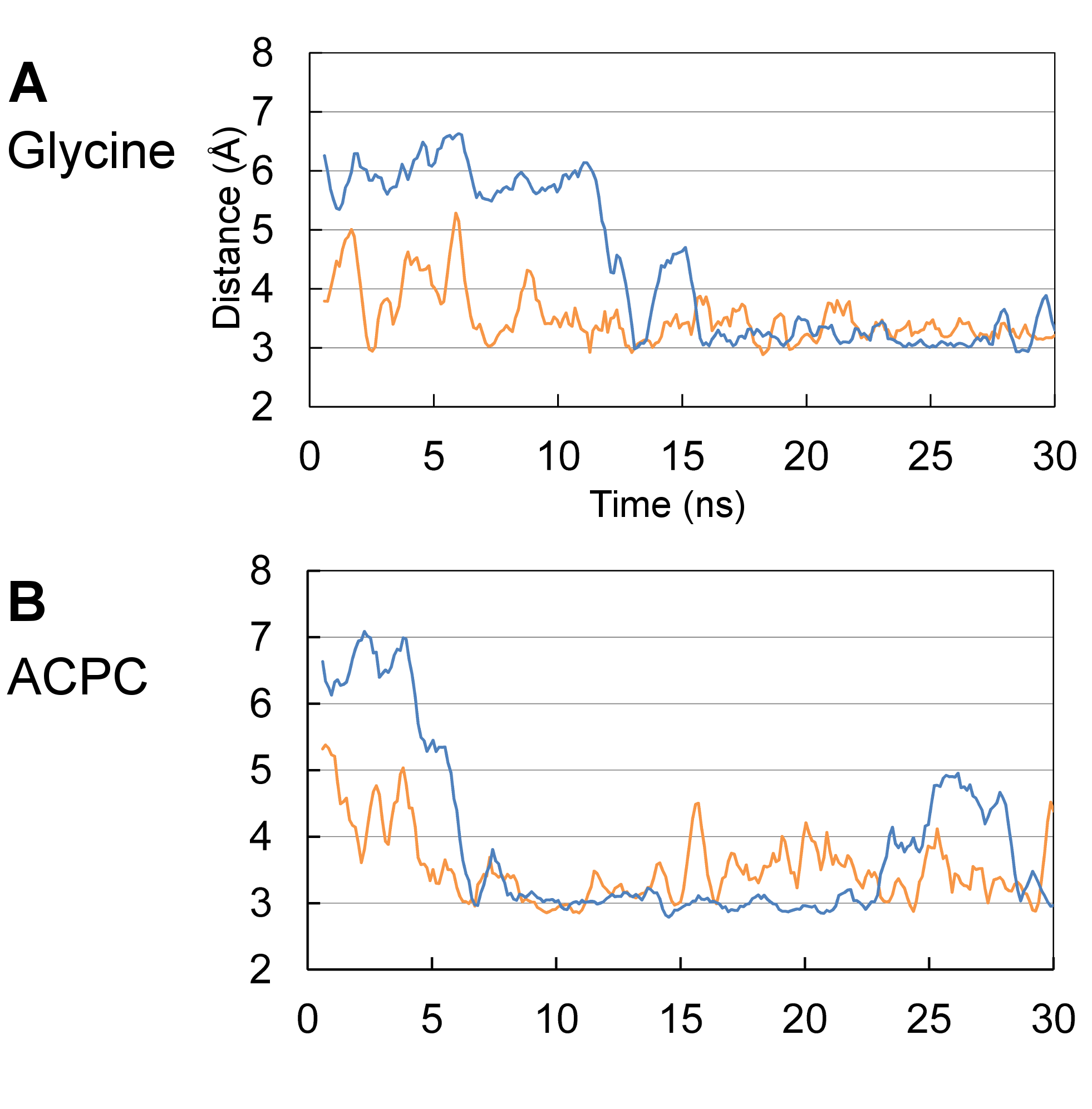

Supplement: Figure S3 — Closure mechanism of glycine and ACPC-bound GluN1-LBD. Distance measurements of Gly458N-Gln686O (blue) and Gln405OE1-Trp731NE1 (orange) from (A) glycine and (B) ACPC-bound open-cleft GluN1-LBD are taken from constraint-free MD simulation trajectories. Similar distance measurements for D-cycloserine bound LBD are shown in Figure 4B. (TIF) [file pone.0047604.s003.tif]

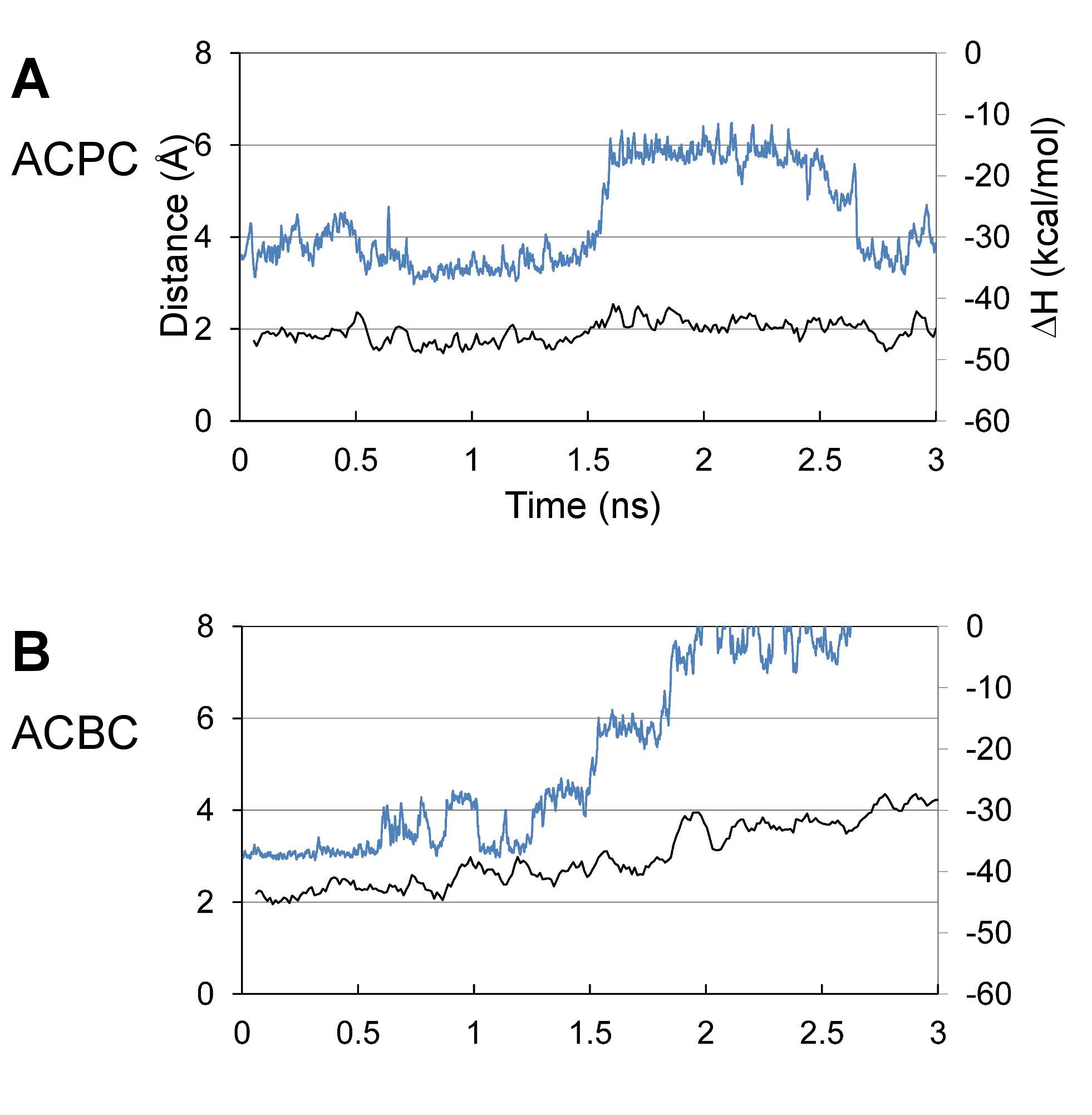

Supplement: Figure S4 — Calculated binding enthalpies (ΔH) from SMD simulations of ACPC and ACBC-bound GluN1-LBD. Compared to D-cycloserine, a similar trend was seen in SMD simulations of (A) ACPC and (B) ACBC: when the LBD opened to an intermediate stage, the ΔH increased only negligibly. With ACPC, this is seen from 1.5 to 2.5 ns and with ACBC, from 1.0 to 1.8 ns. IHB distance is shown in blue and the ΔH, estimated by the MMGB/SA method, in black. (TIF) [file pone.0047604.s004.tif]
